# Alpha-Tocopherol-Infused Flexible Liposomal Nanocomposite Pressure-Sensitive Adhesive: Enhancing Skin Permeation of Retinaldehyde

**DOI:** 10.3390/polym16202930

**Published:** 2024-10-18

**Authors:** Kanokwan Singpanna, Puchapong Jiratananan, Santipharp Paiboonwasin, Nawinda Petcharawuttikrai, Prin Chaksmithanont, Chaiyakarn Pornpitchanarong, Prasopchai Patrojanasophon

**Affiliations:** 1 Pharmaceutical Development of Green Innovations Group (PDGIG), Faculty of Pharmacy, Silpakorn University, Nakhon Pathom 73000, Thailand; 2 Department of Industrial Pharmacy, Faculty of Pharmacy, Silpakorn University, Nakhon Pathom 73000, Thailand; chaksmithanont_p@su.ac.th; 3 Research and Innovation Center for Advanced Therapy Medicinal Products, Faculty of Pharmacy, Silpakorn University, Nakhon Pathom 73000, Thailand

**Keywords:** retinaldehyde, pressure-sensitive adhesive, liposomes, skin permeation, tocopherol

## Abstract

Retinaldehyde (RAL), or retinal, is a vitamin A derivative that is widely used for several skin conditions. However, it is light sensitive and has low water solubility, limiting its efficiency in transdermal delivery. This study developed a novel delivery system for retinal (RAL) using flexible liposomes (FLPs) infused with α-tocopherol succinate (α-TS) to improve stability, and enhance skin permeability. The RAL-FLPs were embedded in pressure-sensitive adhesive (PSA) hydrogels, creating a delivery platform that supports prolonged skin residence and efficient permeation of RAL. The stability and skin permeation as well as human skin irritation and adhesion capabilities were assessed to determine the formulation’s safety and efficacy. Our findings suggested that the addition of α-TS could improve liposomal stability and RAL chemical stability. Moreover, the skin permeation and fluorescence microscopic-based studies suggested that the addition of α-TS could enhance skin permeability of RAL through hair follicles. The RAL-FLP was embedded in PSA hydrogels fabricated from 25% Gantrez^TM^ S-97 (GT) and 1% hyaluronic acid (Hya) with aluminum as a crosslinker. The PSA hydrogel exhibited desirable peeling and tacking strengths. The developed hydrogels also demonstrated greater skin deposition of RAL compared with its aqueous formulation. Additionally, the RAL-FLP-embedded PSA hydrogels showed no skin irritation and maintained better adhesion for up to 24 h compared to commercial patches. Hence, the developed hydrogels could serve as a beneficial platform for delivering RAL in treating skin conditions.

## 1. Introduction

Topical retinoids are commonly used in dermatology for managing conditions such as acne vulgaris and skin aging. Tretinoin, a vitamin A derivative also known as all-trans retinoic acid (RA), is recognized as the most potent topical retinoid. Topical formulations of tretinoin have been approved as a medication for treating acne vulgaris, hyperpigmentation, and facial wrinkles [1]. However, the common side effects of tretinoin include skin irritation, redness, burning sensations, and dryness [2]. Retinoid derivatives are developed with the aim of reducing the harsh side effects. Retinol, retinaldehyde (RAL), and retinyl esters belong to the retinoid family, which are attentively used as active ingredients in skin care products. Among those cosmeceutical ingredients, RAL is outstanding for its low skin irritation profile and high effectiveness [3]. This is because the potent RA is synthesized from all-trans retinol through a two-step oxidative process. In the first step, all-trans retinol is oxidized to RAL, a reaction that is reversible and represents the rate-limiting step in RA biosynthesis. This means that the conversion of retinol to RAL occurs more slowly and regulates the overall pace of RA production. In the second step, RAL undergoes irreversible oxidation to form RA. This reaction proceeds at a higher rate compared to the first step, ensuring that once RAL is produced, it is rapidly converted to RA without the possibility of reversal. Together, these steps highlight the regulatory role of the first oxidation and the efficiency of the second in controlling RA levels [4]. RAL is often considered a preferable precursor to RA compared to retinol due to its faster and irreversible conversion. While retinol’s transformation to RAL is slow and reversible, RAL is more efficiently oxidized to RA, bypassing the rate-limiting step. This ensures a more direct and controlled pathway for RA synthesis, making RAL a more reliable substrate for sustaining RA levels. The irreversibility of RAL oxidation also eliminates the possibility of reversion to retinol, enhancing the overall efficiency of RA production [5,6].

For acne treatment, RAL helps reduce sebum production, clear clogged pores, inhibit bacterial growth, and is also effective in managing post-inflammatory hyperpigmentation [7]. Considering the skin rejuvenation, RAL is converted to RA through RAL dehydrogenase which stimulates collagen production and promotes cell turnover, thereby reducing fine lines, wrinkles, and hyperpigmentation. Consequently, RAL has become a popular choice for anti-aging [1]. Nevertheless, RAL application is limited by poor water solubility, making incorporation into an aqueous vehicle difficult [8]. Additionally, RAL is highly unstable and easily undergoes auto-oxidation and photo-oxidation [9]. Continual research efforts are currently underway to enhance the stability and efficacy of the product through the implementation of various approaches, including the use of nanotechnology.

Innovative formulations utilizing nanotechnology are currently in development and undergoing testing to enhance the stability of the active ingredient, increase its skin penetrability, control its release from the formulation, and achieve optimal efficacy while minimizing skin irritation and other adverse effects [10,11,12]. The use of nanotechnology in skin care products is rapidly expanding in the cosmetic industry. Lipid-based delivery systems are the most popularly used due to numerous advantages over other nano-systems, notably regarding their low toxicity, biodegradability, potential for large-scale manufacturing, and the wide range of chemical and formulation options [13,14,15]. Various lipid-based nanocarriers include liposomes, niosomes, solid lipid nanoparticles (SLNs), nanostructured lipid carriers (NLCs), and nanoemulsions. For instance, a combination of 0.05% RAL and coenzyme Q10 encapsulated in NLC gel-based formulations effectively reduces wrinkles with minimal skin irritation in animal models [16]. RAL-loaded niosomes have been clinically proven to be effective in improving acne after 4 weeks and safe for human subjects. Participants with mild-to-moderate acne showed a significant decrease in comedones and sebum secretion, as well as an improvement in skin desquamation. Additionally, human subjects tolerated RAL-loaded niosomes well, with no reported skin irritation or serious adverse effects [17].

The nanocomposites are a novel strategy that integrates nanoparticles (NPs) within a matrix material such as polymers [18,19]. There is a growing interest in utilizing nanocomposite transdermal patches to enhance transdermal delivery capabilities, such as in controlled drug release, increasing drug carrying capacity, improving residence time, and improving the precision of drug targeting [20]. Particularly, with pressure-sensitive adhesive (PSA) patches, adhesive materials that can bond to a surface with light pressure demonstrate superior adhesion capability in comparison to gels and creams, thereby lessening the necessity for frequent reapplication. The PSA patches improve user compliance with ease in application as they typically do not rely on a liquid-to-solid transitions to secure materials together and only require slight force, e.g., applying finger press force. In cosmetic industries, biopolymers are commonly used due to their safety and biocompatibility [21]. In the development of a transdermal patch, the utilized materials must demonstrate robust adhesion, skin compatibility, and minimal potential for skin irritation to optimize user compliance and comfort [22]. Hyaluronic acid (Hya) is a natural polymer composed of repeating disaccharide units: *N*-acetyl-D-glucosamine and D-glucuronide. It is primarily found in body fluids and tissues, especially in the skin. Due to its biocompatibility and non-immunogenic properties, Hya is widely used in pharmaceutical and cosmeceutical hydrogels. Additionally, in terms of mechanical properties, Hya also provides appropriate flexibility and strength for skin application [23]. Gantrez^TM^ S-97 (GT) is a synthetic copolymer containing alternating units of methylvinylether and maleic anhydride. It has been widely used for pharmaceutical purposes as a thickening and suspending agent, as well as an adjuvant for transdermal patches for decades [24]. GT provides exceptional adhesion and flexibility, making it a versatile and reliable option for creating long-lasting and effective transdermal patches.

These materials have the potential to produce safe, effective, and comfortable transdermal hydrogel patches for long-term use. Furthermore, when combined with biocompatible lipid-based NPs, this approach can provide a more efficient and safer alternative to traditional delivery methods. This study focused on the development of RAL entrapped in flexible liposomes (FLPs) that was infused with α-tocopherol succinate (α-TS) to enhance stability and skin permeability, while lessening skin irritation. The FLPs were incorporated into adhesive hydrogels which presented as an effective FLP delivery platform that facilitated skin residence and permeation. Additionally, the study assessed adhesive performance and in vitro drug penetration in the skin, as well as examined skin irritation and adhesion capability in human subjects. The findings presented a novel approach to enhancing the stability and skin permeability of RAL via nanocomposite pressure-sensitive adhesive hydrogel, validated for human safety.

## 2. Materials and Methods

### 2.1. Materials

RAL was purchased from Chanjao Longivety Co., Ltd. (Bangkok, Thailand). Egg phosphatidylcholine (PC) (Phospholipon^®^ 90G) was purchased from Lipid GmbH (Ludwigshafen, Germany). Cholesterol from lanolin (purity 95%) was purchased from Fluka (Tokyo, Japan). D-α-Tocopherol succinate (α-TS) and Triton^®^ X-100 were supplied from Sigma-Aldrich (St. Louis, MO, USA). Lissamine™ Rhodamine B 1,2-dihexadecanoyl-sn-glycero-3-phosphoethanolamine, triethylammonium salt (Rhodamine DHPE) was purchased from Thermofisher Scientific (Waltham, MA, USA). Poly(methyl vinyl ether-alt-maleic acid) copolymer (Gantrez^®^ S-97, MW 1500 Da) was purchased from Ashland Inc. (Surrey, UK). Hyaluronic acid (Hya, MW 1200–1800 kDa) was purchased from P.C. Drug Center (Bangkok, Thailand). Aluminum glycinate and DL-malic acid were purchased from Tokyo Chemical Industry Co., Ltd. (Tokyo, Japan). All other chemicals were commercially obtained and used as received. Porcine skin was collected from neonatal pigs that died naturally at Charnchai Farm in Ratchaburi, Thailand.

### 2.2. Development of Retinal-Loaded Flexible Liposomes

#### 2.2.1. Preparation of Liposomes

Firstly, stock solutions of PC, cholesterol, and α-TS were prepared by dissolving in a chloroform/methanol (2:1 *v*/*v*) solvent mixture. PC stock solution was prepared by dissolving 0.773 g of in 5 mL of the solvent mixture (200 mM); cholesterol stock solution was prepared by dissolving 0.062 g in 8 mL (20 mM), and 0.085 g of α-TS was dissolved in 8 mL of solvent to create α-TS stock solution (20 mM). The liposome formulations were prepared using thin-film hydration and sonication methods. The process was carried out in a darkroom to prevent photodegradation of the active ingredient. Two distinct liposomal formulations were prepared, which were the flexible liposome (FLP) of α-TS -infused liposomes and conventional liposomes. To prepare the α-TS-infused FLP, 100 µL of PC and 200 µL of α-TS stock solution (10:2) were added to a test tube and mixed using a vortex mixer. The solvent was evaporated using a nitrogen stream, while the tube was slowly rotated to create the thin film. The lipid film was placed in a desiccator and dried overnight. Then, 5 mL of phosphate-buffer saline (PBS pH 7.4) was added into the test tube and mixed with the lipid film using a vortex mixer. To reduce the particle size of the FLPs, the obtained mixture was sonicated for 30 min using a 40 kHz probe ultrasonicator at 40% amplitude (Vibra-Cell^TM^, Sonics, and Materials, Newtown, CT, USA). After that, the resulting solution was centrifuged at 15,000 rpm at 4 °C for 15 min to eliminate the excess lipid and unentrapped drug. Finally, the supernatant containing FLPs was collected for further study. The conventional liposomes were prepared using the same procedure, except that cholesterol was used instead of α-TS.

#### 2.2.2. Characterization of Retinal-Loaded Flexible Liposomes

The particle size, polydispersity index (PDI), and zeta potential (ZP) of the liposomes were determined using dynamic light scattering (DLS) (Zetasizer Nano ZS, Malvern, UK). The measurements were conducted in triplicate at a 90° angle and 25 °C. To observe the morphology of liposomes, a transmitted electron microscope (TEM) was used for visualization (Philips TECNAI 20, Hillsboro, OR, USA).

#### 2.2.3. Drug Loading

The RAL-loaded FLPs (RAL-FLPs) and RAL-loaded conventional liposomes (RAL-LPs) were prepared by adding RAL at various concentrations (1, 3, and 5 mg/mL) to the organic phase prior to preparing the thin film; other processes were conducted as aforementioned. The RAL content was quantified using high-performance liquid chromatography (HPLC). The liposomes were disrupted in Triton^®^ X-100 at a ratio of 1:1 and diluted in methanol prior to quantification. The percentage of drug loading capacity (%LC) and percentage of drug entrapment efficacy (%EE) were calculated using Equations (1) and (2), respectively.

(1) $$\%\mathrm{LC}=\frac{\mathrm{Quantified}\;\mathrm{RAL}\;\mathrm{content}}{\mathrm{Total}\;\mathrm{lipid}\;\mathrm{content}}\times \;100$$

(2) $$\%\mathrm{EE}=\frac{\mathrm{Quantified}\;\mathrm{RAL}\;\mathrm{content}}{\mathrm{Initial}\;\mathrm{RAL}\;\mathrm{content}\;\mathrm{added}}\times 100$$

#### 2.2.4. Stability Study of Retinal-Loaded Flexible Liposomes

The liposome formulations were kept in the dark at 4 °C. At each time point up to 21 days, the stability of RAL-LPs was evaluated by measuring their physicochemical characteristics and RAL content using a zetasizer and HPLC, respectively.

#### 2.2.5. HPLC Analysis

RAL content analysis was conducted using an HPLC system (Agilent 1220, Agilent Technologies Inc., Santa Clara, CA, USA) equipped with a reversed-phase column (C18, 4.6 mm × 25 cm; 5 µm). Methanol was used as the mobile phase at the flow rate of 2 mL/min. The injection volume was 20 μL. The detection was performed using a UV detector at a wavelength of 325 nm. The amount of RAL in the samples was calculated using a standard curve that showed excellent linearity across the concentration range of 1–500 μg/mL. The R^2^ of the mean calibration plot was 0.9999.

#### 2.2.6. In Vitro Skin Permeation Study of Retinal-Loaded Liposome

The abdominal skin area of the neonatal pig was obtained and used as a model membrane in this study. The subcutaneous fat layer was dissected with scissors and a surgical blade. The skin was kept at −20 °C until use. In vitro skin permeation study of RAL-FLPs and RAL-loaded conventional liposome (RAL-LP) was conducted using vertical Franz diffusion cells. The experiment was set up as follows. First, the porcine skin was thawed in PBS pH 7.4 at room temperature and placed between the donor and receptor compartment containing 6 mL of PBS/Isopropyl alcohol (1:1) and a stir bar. The receptor compartments were connected to the water circulator bath to maintain the temperature of the skin surface at 32 ± 2 °C. Then, 1 mL of RAL-FLPs or RAL-LPs was added into the donor compartment and covered with aluminum foil to prevent photodegradation of the RAL. At predetermined time points, 0.5 mL of the receptor solution was collected for drug analysis. To keep the volume constant, the same quantity of new medium was added back in at each sampling time. The samples collected for analysis were subjected to filtration through a 0.45 μm syringe filter prior to HPLC analysis. The cumulative drug that permeated through the skin was plotted against time, and the transdermal flux at steady state (J) was computed from the slope of the graph. The permeability coefficient (K_p_) of the drug was calculated by employing Fick’s law of diffusion model, as shown in Equation (3). The liposomal formulations with and without α-TS were compared, and the RAL solution was used as a control.

(3) $$K_{p}=\frac{J}{C_{d}}$$

where C_d_ is the concentration of the drug in the donor compartment.

#### 2.2.7. Confocal Laser Scanning Microscope Study

The skin penetration pathway of RAL-FLPs was studied by confocal laser scanning microscope (CLSM) using the co-localization technique. The liposome particles were labeled with Rhodamine DHPE, a red fluorescent phospholipid, by adding to the lipid film during the liposomal preparation process, as described in Section 2.2.1. The skin permeation study was performed using Franz diffusion cells, as described in Section 2.2.6. The donor part was filled with 1 mL of rhodamine DHPE-labeled RAL-FLPs. After 4 h, the skin was collected and washed with PBS to remove the excess drug. To perform CLSM imaging (Zeiss LSM 800 Airy scan, Carl Zeiss, Jena, Germany), the skin sample was mounted on a glass slide with the stratum corneum facing the objective lens using methyl salicylate as an immersion oil. The CLSM images of the area of interest were captured sequentially in the x–z plane and processed by ZEISS ZEN2 (Blue edition) software version 3.10. The images showed the rhodamine DHPE-labeled liposomal particles in red and the RAL in fluorescent green.

### 2.3. Development of Retinal-Loaded Flexible Liposome-Embedded Pressure-Sensitive Adhesives

#### 2.3.1. Preparation of Pressure-Sensitive Adhesive Patch

The PSA patches were fabricated from GT and Hya using aluminum glycinate as a crosslinker and malic acid as a crosslinking regulator. GT and Hya were prepared by dissolving in deionized water. Separately, aluminum glycinate and malic acid was dispersed in glycerin. The glycerin mixture was then combined with the polymer mixture, and mixed until homogeneous. The adhesive mixture was casted onto a 3.5 × 5 cm backing fabric, and dried overnight at ambient temperature. The PSA patches were developed by varying the concentration of the PSA hydrogel formers, as listed in Table 1. The RAL-FLP colloidal dispersion was added to the PSA patch to contain RAL equivalent to 0.05% *w*/*w*.

#### 2.3.2. The Evaluation of PSA Patches

##### Probe Tack Test

The measurement of the force required to detach a probe from an adhesive at a constant speed is known as tack strength. In this study, the probe tack test was used to determine the tacking strength of the PSA patches. A texture analyzer (TA.XT Plus, Stable Micro Systems, Hamilton, OH, USA) connected with a cylindrical Perspex probe (P 0.5/R) and a 5 kg load cell were employed for the measurement. The probe was set to move to contact with the adhesive surface at a speed of 1 mm/s and compressed with a force of 5 N. Then, the probe was steadily pulled away at a speed of 0.5 m/s. The maximum force required to detach the probe from the surface was recorded, and the measurement was conducted in triplicate.

##### Peel Adhesion Measurement

Peel adhesion force refers to the force required to remove an adhesive from a surface in a horizontal direction. This characteristic is particularly significant since the adhesives must be taken off without harming the skin or leaving any residue. A texture analyzer (TA.XT Plus, Stable Micro Systems, Hamilton, OH, USA) was used with a tensile grip holder. The patch was attached to a testing platform on one end and held by a grip holder on the other end. The patch was then pulled at a speed of 2 mm/s in a direction opposite to the test platform at an angle of 180 degrees. The maximum force required to remove the patch from the platform was recorded. The measurement was carried out in triplicate.

##### Drug Release Study

The study was performed to illustrate the drug release profile from the PSA patches. The patches that contained RAL-FLPs were cut into pieces measuring 1 × 1 cm. These pieces were then placed in a solution of 2% Tween 80 in PBS (pH 7.4) and transferred to an incubator shaker operating at a temperature of 37 ± 2 °C with a shaking speed of 75 rpm. At specific predetermined time points, 0.5 mL of release medium was withdrawn and analyzed for drug content using HPLC.

#### 2.3.3. Skin Deposition Study

The investigation into RAL skin deposition utilized vertical Franz diffusion cells. The PSA patches, which contained RAL-FLPs, were cut into 1 × 1 cm pieces and then applied to the skin at the donor site. As a control, a RAL aqueous solution was used. The experimental setup is detailed in Section 2.2.5. After 24 h, pig skin samples underwent cleaning and wiping with PBS pH 7.4 to eliminate excess drug material on the skin surface. Following this, the skin diffusion area was cut into small pieces and immersed in a 1:1 mixture of methanol and PBS. The mixture was then vortexed for 10 min and incubated in a shaker at 37 °C for 24 h. Subsequently, the solution was collected and centrifuged at 12,000 rpm at 4 °C for 15 min. Before drug content analysis by HPLC, the samples were filtered through a 0.45 µm filter. Calculations for drug accumulation in the skin were made per unit area of the skin (µm/cm^2^).

#### 2.3.4. In Vivo Skin Irritation Study and Adhesion Capability in Human Subjects

The examination on skin irritation was conducted on a group of 25 healthy human volunteers aged between 25 and 40 years old. This study was approved by the Human Studies Ethics Committee of the Research, Innovation and Creativity Administration Office, Sanam Chandra Palace Campus, Silpakorn University (Approval No: REC 67.0126-014-0505). The participants committed to refraining from using any topical items or consuming antihistamine medication prior to the study. RAL-loaded liposome-embedded PSA, blank PSA, and commercial patches were cut into 2 × 2 cm in size, applied at the inner region of the upper arms on both sides, and covered with waterproof adhesive films. After 24 of the application, the redness of the skin was measured as the erythema index (EI) using a skin analysis system (DermaLab^®^ series, SkinLab Combo, Aalborg, Denmark). The EI value was assessed before (*E*) and after 24 h of patch application (*E_t_*). The percentage erythema index (%EI) was calculated by comparing the EI of the skin region of interest to that of normal skin using Equation (4). In addition to skin irritation, adhesion capability was also assessed using the same procedure but without waterproof adhesive film. The time that patches remained attached to the skin was recorded.

(4) $$\%\mathrm{EI}=(100+\frac{E_{t}-E}{E})\times 100$$

### 2.4. Statistical Analysis

The experimental data are reported as mean ± standard deviation (SD). The statistical analysis was conducted using the *F*-test and independent *t*-test for comparison of the 2 groups, while ANOVA followed by Tukey’s post hoc test was used to distinguish the difference among other comparisons. A statistical difference is indicated when the *p*-value is less than 0.05.

## 3. Results and Discussion

### 3.1. Development of Retinal-Loaded Flexible Liposomes

#### 3.1.1. Characterization of Liposomal Formulations

**Figure 1:**
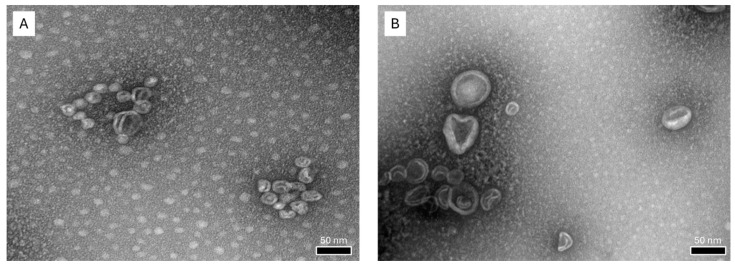
TEM images of RAL loaded in (**A**) α-TS-FLPs and (**B**) conventional LPs (at a magnification of 175 k×).

Conventional liposomes are the first-generation liposomes. Their major components include natural phospholipids, such as PC, as the primary components of the liposomal membrane and cholesterol, which aid in stabilizing the membrane and improving vesicle fluidity [25]. In this study, two different liposomal formulations were compared. One was conventional liposomes composed of PC and cholesterol, while the other used α-TS as a stabilizing agent. Table 2 displays the physicochemical characteristics of liposomes, comprised of average particle size, PDI, and ZP. The liposomes containing α-TS had a smaller particle size compared to conventional liposomes. Based on TEM images (Figure 1), both liposomes exhibited a nearly spherical morphology. Moreover, both liposomal formulations had a narrow size distribution of PDI value less than 0.3 indicating a considerable homogeneous particle size distribution. The FLPs containing α-TS had a negative surface charge with ZP value, whereas the surface charge of conventional liposomes is nearly neutral. Since the succinate ester form of α-tocopherol was used in the formulation, the ionized succinate group is responsible for the negative surface charge by the carboxylic group presented. α-TS shares structural similarities with surfactants due to its amphiphilic nature. Like surfactants, it has both hydrophilic and hydrophobic regions. The hydrophobic portion is the tocopherol (vitamin E) component, while the succinate group introduces hydrophilic properties. This dual characteristic allows α-TS to interact with both lipid and aqueous environments, similar to how surfactants stabilize the FLP structure as reported in [26]. These structural features make it capable of playing roles in stabilizing the liposomal vesicles, as well as improving its flexibility, creating FLPs to enhance skin permeation. A similar attempt has been made by Park et. al. (2023), which utilized ergosterol esters containing unsaturated fatty acids to form a lipid bilayer with PC. The study suggested that ergosterol esters have an important effect on membrane flexibility [27]. Thus, the use of α-TS presenting negative ZP could play an important role in enriching colloidal stability of the liposomes.

#### 3.1.2. RAL Loading

Various concentrations of RAL were added to the liposome formulation to examine the suitable condition for drug loading. According to Table 3, R1 presented the smallest particle size with homogeneous size distribution and similar ZP compared to the other drug loading conditions. Moreover, the %LC and %EE of R1 were the highest amongst the formulations (*p* < 0.05). This could be due to the tiny liposomal vesicles limiting the amount of RAL able to reside at the lipophilic layer leading to the maximum capacity found at 55% of RAL compared to total lipid content. The amount of RAL was varied in order to find the most suitable amount of RAL to be loaded into the FLPs. Meanwhile, the highest RAL content was expected as this would facilitate the fabrication of RAL-FLP-embedded PSA hydrogels with tunable efficacious RAL content for skin rejuvenation. It was found that the concentrations of RAL varied yielded a predictable trend that with higher initial amount of RAL, the %EE decreased significantly. Therefore, the RAL initial concentration of 1 mg/mL was the most feasible amount of RAL to form desirable RAL-FLPs. Furthermore, the stabilizing FLPs using α-TS could play a role in maximizing the drug content as formerly reported with cholesterol-containing liposomes [28]. This indicated that RAL loading with the initial RAL concentration of 1 mg/mL was the most appropriate for RAL-FLP preparation. Hence, this condition was selected for the preparation of RAL-FLPs and RAL-LPs for further experiments.

When the initial RAL content of 1 mg/mL was applied to the preparation of RAL-LPs, it was found that the %LC and %EE were 50.25 ± 0.54 and 52.86 ± 0.25, respectively. These findings suggested that the RAL encapsulation capabilities were similar to the RAL-FLP formulation (*p* > 0.05).

#### 3.1.3. Stability Study of Retinal-Loaded Liposomes

**Figure 2:**
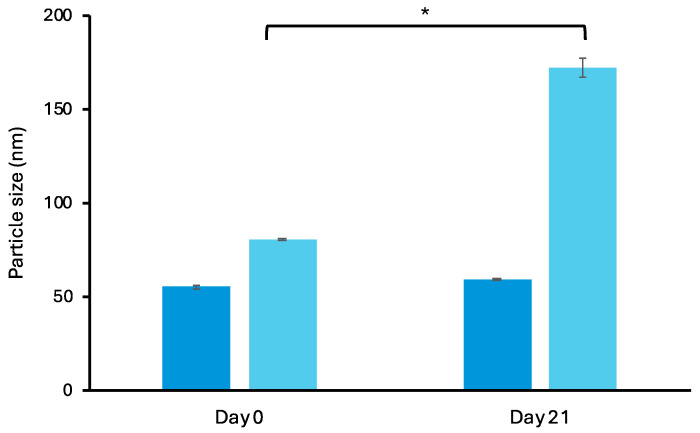
Particle size of (■) α-TS-FLPs and (■) conventional LPs at day 0 and 21. * Significant difference vs day 0 (*p* < 0.05).

The colloidal stability of conventional LPs and α-TS-FLPs was assessed on day 0 and 21. Figure 2 shows no significant change in particle size from α-TS-FLPs, indicating particle stability of α-TS-FLPs. Meanwhile, the particle size of conventional LPs significantly increased after 21 days. Particle stability and aggregation are influenced by the properties of their surfaces. Electrostatic repulsion arising from either a positive or negative charge on particle surfaces plays a critical role in particle stabilization and preventing aggregation. The larger ZP value can lead to higher repulsing force between particles, and thus the stability increases [29]. From the literature, formulations that have a ZP of ±30 mV or more influence the colloidal stability of nanoformulations [30]. Therefore, it is suggested that the better particle stability of α-TS-FLPs (compared with conventional LPs) could be related to the higher negative charge presented by the succinyl group of α-TS.

**Figure 3:**
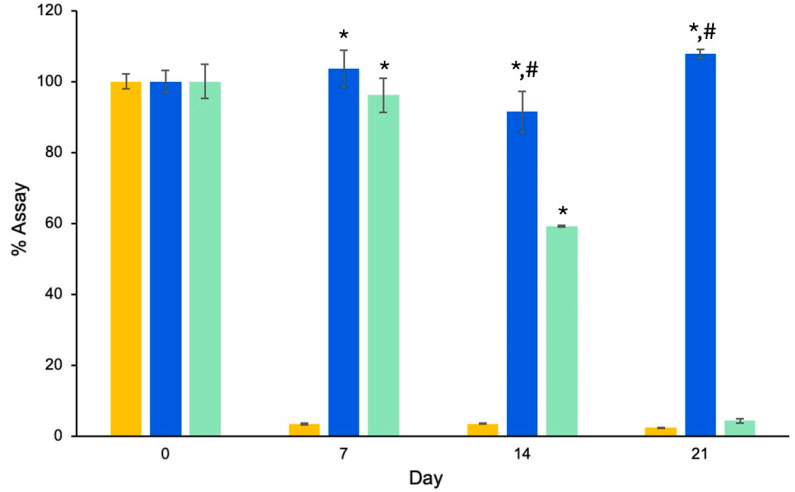
Percent assay of RAL content in (■) RAL-FLPs after 7, 14, and 21 days, compared to (■) RAL-LPs and (■) RAL solution (control) (Significant difference from control (*) and RAL-LPs (^#^), *p* < 0.05).

Figure 3 shows the chemical stability of RAL-FLPs compared to RAL-LPs and RAL aqueous solution. The results are expressed as the %assay. In solution form, RAL degraded rapidly from 100% on the first day to 3.39% after 7 days. The observed phenomenon may be attributed to the transformation of RAL to retinoic acid through the oxidation process [31]. In addition to enhancing drug delivery, liposomes are also used to preserve the internal contents [32]. In our study, α-TS-LPs could preserve the entrapped RAL for up to 21 days with no significant changes (*p* > 0.05), while the conventional liposomes could prevent the degradation to only 7 days.

Owing to the antioxidation properties, α-tocopherol (or vitamin E) in pharmaceutical or cosmetic formulations plays a vital role in protecting the formula and active ingredients from oxidation [33]. It is recommended that α-tocopherol be utilized to improve the stability of cosmetic ingredients prone to degradation, such as vitamin C, vitamin A, and their derivatives. Ascorbic acid (or vitamin C) is an ingredient that is extensively used for anti-aging cosmetic products. The rapid degradation of the ascorbic acid is influenced by oxygen, light, high temperature, the pH of the medium, and metal ions such as Cu^2+^, Fe^2+^, and Zn^2+^, which poses a major problem for formulation [34,35]. Numerous reports have highlighted the effectiveness of combining ascorbic acid with α-tocopherol in preserving the stability of ascorbic acid [36,37]. Moreover, various studies have indicated that the incorporation of α-tocopherol in the formulation has the potential to extend the shelf-life of vitamin A in creams. The study from Guaratini et al. (2006) suggested that the addition of the antioxidant α-tocopherol could extend the shelf-life of vitamin A palmitate to 77 days [38]. The stability improvement of these active compounds could be due to an intermolecular interaction with α-tocopherol that reduces the reactivity of molecular oxygen [39].

The findings of the current study are consistent with existing literature, indicating that the preservation of RAL can be ascribed to the antioxidant properties of α-tocopherol. Moreover, the incorporation of RAL into the FLP vesicles improved the stability of the active compound. The FLPs enhance stability and provide protection from photo-oxidation by encapsulating RAL within their phospholipid bilayer. The lipid bilayer acts as a physical barrier, shielding the encapsulated substance from environmental factors such as light, oxygen, and reactive species that cause photo-oxidation. Therefore, the improved stability of RAL was proposed to be responsible by the vesicle and infused α-TS.

#### 3.1.4. In Vitro Skin Permeation of Retinal-Loaded Liposomes

**Figure 4:**
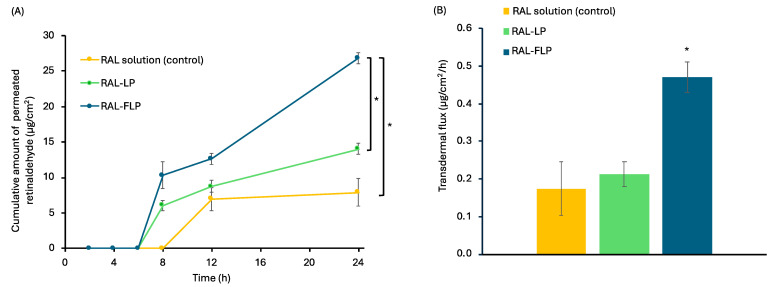
(**A**) Skin permeation profile and (**B**) transdermal flux at steady state of RAL in solution (control), conventional LPs, and α-TS-FLPs. Each data point represents the mean ± SD (n = 3) (* significant difference vs control, *p* < 0.05).

The assessment of percutaneous drug permeation is a vital parameter in the evaluation of topical drug delivery systems. Figure 4A,B illustrate the skin permeation profile and transdermal flux at the steady state of RAL-FLPs, conventional LPs, and RAL aqueous solution (control). Among the three groups, the highest skin permeation of RAL was observed from α-TS-FLPs, followed by the conventional LP and the RAL solution, respectively. This is represented by the cumulative drug permeation across the model membrane. Moreover, the diffusion rate indicated by permeation flux proved that the RAL-FLPs influence the transdermal delivery of RAL. In an aqueous solution, the transdermal flux of RAL was 0.17 ± 0.07 g/cm^2^/h. Incorporating RAL into α-TS-FLPs significantly increased transdermal flux to 2.7 times (0.47 ± 0.04 g/cm^2^/h, *p* < 0.05), whereas conventional LPs could slightly improve the skin permeation of RAL (0.21 ± 0.03 g/cm^2^/h).

Vitamin A derivatives are a group of lipophilic compounds, including retinol, RAL, and retinyl esters. Due to their highly lipophilic nature, vitamin A-derivative molecules have low percutaneous absorption [40,41]. According to the present study, the limited skin permeation of the free RAL in an aqueous solution could be due to its highly lipophilic profile (logP = 6.62). The encapsulation of these molecules into nanocarriers is an attractive strategy for improving stability and skin permeation [42]. Because of their small size, nanocarriers can penetrate the skin more easily. Various mechanisms of liposomes have been proposed. Liposomes may induce a structural change in the lipid matrix, or they may accumulate in the appendageal space and release the encapsulated active ingredients. Due to the similar lipid composition between liposomes and the skin’s lipid structure, the liposome bilayer could fuse with the stratum corneum and result in enhanced permeation of the encapsulated active ingredients [12,43]. Likewise, the present study suggests that the encapsulation of RAL in α-TS-FLPs could greatly improve skin permeation of the lipophilic active compounds. Pena-Rodriguez et al. (2020) also demonstrated an increased skin penetration of vitamin A derivative, retinyl palmitate, when encapsulated in the flexible lipid-based vesicular nanocarrier, transfersomes [40]. Furthermore, Limcharoen et al. (2019) revealed that the incorporation of RAL in polymeric nanocarriers could lead to higher concentrations in the stratum corneum and hair follicles, compared with the free RAL in solution. This result suggests that incorporating RAL in nanocarriers enhances the permeation of active ingredients into the skin [10].

The enhanced transdermal permeation of RAL could be attributed not only to the implementation of nanocarriers but also to the substantial impact of α-tocopherol. According to our findings, the infusion of α-tocopherol into liposomes resulted in an approximately 2-fold increase in the transdermal flux of RAL compared to the conventional LPs, rising from 0.21 to 0.47 g/cm^2^/h. From the literature, α-tocopherol can intercalate within the lipid bilayer domain of the stratum corneum, which may disrupt the gel-phase lipid, and alter membrane permeability [44,45]. Therefore, significant improvement of skin permeation was observed when using nanocarriers together with α-tocopherol. Furthermore, because of its negatively charged nature and long-chain structure, it is possible that α-TS could influence the flexibility of liposome vesicles and potentially result in increased skin permeation similarly to that was reported on ergosterol ester [27].

#### 3.1.5. CLSM Study

**Figure 5:**
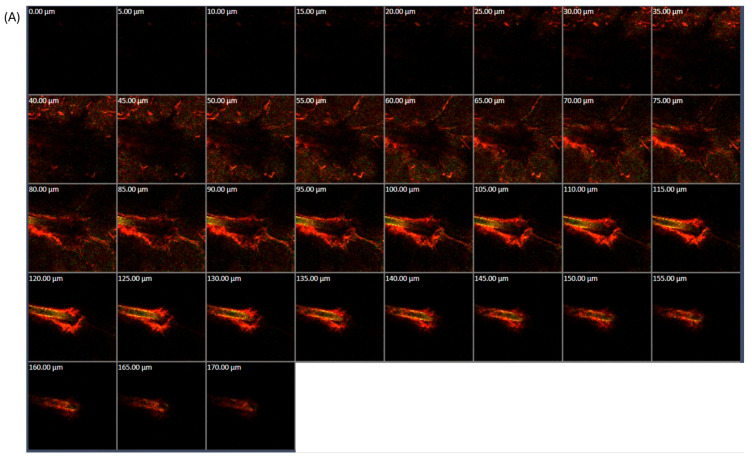

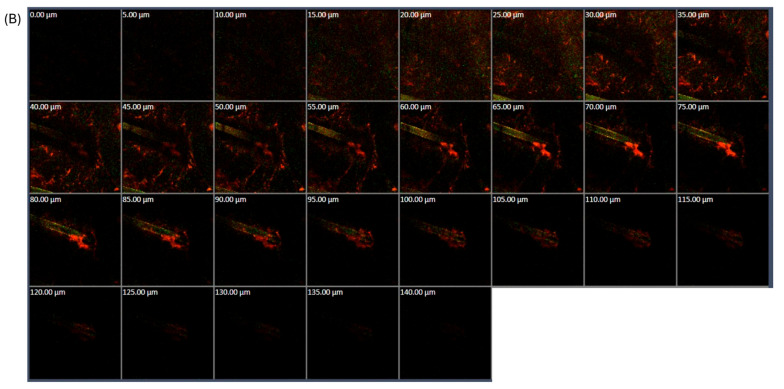
Two-dimensional CLSM images of RAL skin penetration when encapsulated in (**A**) α-TS-FLPs and (**B**) conventional LPs near hair follicles after 4 h of treatment.

To investigate the penetration mechanism of liposomal formulations, CLSM was employed. The distribution of RAL and liposomes within the skin layers was traced and visualized. Liposome vesicles were labeled with DHPE, causing them to exhibit a distinctive red fluorescence under CLSM, while RAL was characterized by a yellowish-green fluorescence. From Figure 5, a red fluorescent signal was observed at the hair follicle area for both liposome formulations, conventional LPs and α-TS-FLPs. Moreover, it was noted that α-TS-FLPs exhibited deeper follicular penetration and higher intensity of red and yellowish-green fluorescence signals with the deepest permeation of 170 µm (Figure 5A), while the deepest penetration of conventional LPs was at 140 µm (Figure 5B).

Several potential mechanisms for how liposomes enhance skin penetration have been proposed. Firstly, the free drug permeates after its release from the carriers. Secondly, the components of liposomes act as permeation enhancers to facilitate permeation. Thirdly, liposomes can fuse with the stratum corneum, thereby facilitating drug permeation. Fourth, liposomes loaded with drugs are capable of penetrating directly through the skin. Fifth, the drug and nanovesicle are transferred though transappendageal permeation [43]. The red and yellowish-green fluorescence surrounding the hair follicle in our findings suggested the fifth mechanism of liposome action, indicating the penetration pathway through the transappendageal route. In addition to liposomes, other types of nanocarriers also utilize the transappendageal route as their penetration pathway. A study reported that polymeric nanoparticles fabricated from chitosan, with a particle size of 249 nm, demonstrated the ability to penetrate the follicular region as well [10]. Therefore, the transappendageal route is considered the most common pathway for nanocarriers.

The efficiency of skin permeation by liposomes can be affected by several factors, including lipid composition, particle size, surface charge, flexibility, elasticity, and the type of liposomes [46]. Based on our findings, Figure 5A illustrates a greater skin permeation of RAL-FLPs through hair follicles compared with conventional LPs (Figure 5B). The enhanced penetration can be attributed to multiple factors related to the structural and functional properties of the liposomal formulation. The study shows that α-TS-FLPs achieved greater accumulation in hair follicles compared to conventional liposomes (Figure 5A). This is a critical finding because hair follicles are known to serve as effective reservoirs for drug delivery, providing a direct route for deeper skin permeation. The smaller particle size of the α-TS-FLPs, as observed, plays a crucial role in facilitating this permeation. Liposomes with reduced particle sizes can more efficiently penetrate the narrow openings of hair follicles, making this system highly effective for targeting transfollicular pathways. This contrasts with larger liposomes, which may have more difficulty navigating the follicular structures (Figure 5B). One of the standout factors in the improved skin permeation of α-TS-FLPs is the role of α-TS. This compound likely increases the flexibility and fluidity of the liposomal bilayers, which enhances their ability to deform and pass through the complex skin layers, including the narrow follicular channels. The liposomal bilayers become more malleable, allowing the vesicles to adapt to the structural demands of the skin’s barrier and appendages. This flexibility is essential in increasing the overall effectiveness of skin delivery, as liposomes with enhanced deformability can penetrate deeper into the skin than rigid structures. In addition to its contribution to liposome flexibility, α-TS is known for its permeation-enhancing properties. By altering the lipid environment of the stratum corneum and potentially disrupting the lipid matrix, α-TS may facilitate the penetration of both the liposome and RAL. This dual action of improving liposome flexibility and enhancing permeation through the skin barrier results in a synergistic effect, maximizing the delivery of RAL to the targeted skin layers [47].

This result suggested that the integration of α-TS increased the accumulation of FLPs in hair follicles. Regarding the particle size examined, the α-TS-FLP was relatively smaller than the conventional LP, facilitating the permeation through hair follicles. The enhanced follicular permeation of α-TS-FLPs could also be explained by the second mechanism, as α-tocopherol is known to enhance skin permeation [45]. Moreover, α-tocopherol may play a role in altering the flexibility and deformability of liposomes, which could allow for easier penetration. The skin permeation enhancement of α-TS-FLPs may also be the result of synergistic mechanisms. However, further investigation is required to determine the exact mechanism.

### 3.2. Development of Retinal-Loaded Flexible Liposome-Embedded Pressure-Sensitive Adhesive Hydrogels

#### 3.2.1. Characterizations and Release Study of Pressure-Sensitive Adhesive Hydrogels

Five different formulations of hydrogel adhesives were tested for their mechanical properties, particularly tacking strength and peel adhesion force, which are critical parameters for evaluating the effectiveness of adhesive materials. The results demonstrate notable differences in the adhesive properties of the various formulations. For formulations F1 and F3, the appearance of the patches can be described as “soft and adhesive” (Table 4), and they exhibited moderate to high tack strengths, respectively. These values suggest that these formulations maintain strong adhesive properties without leaving residue, making them potentially suitable for applications requiring easy removal and strong adhesion. Thus, F5 and F3 exhibited the highest tacking and peeling adhesion force. This is likely attributable to the higher polymer content, endowing it with robust adhesion properties. However, the major drawback of F5 was the stain and polymer residues that remained after it was removed. Formulation F3 was composed of a higher concentration of GT and a lower concentration of HA compared to F5. Despite its slightly softer texture, F3 demonstrated comparable adhesive properties to F5. Considering the peeling force, lower peeling force may be desired for making an easy-to-peel patch. But, a trade-off between adhesion strength and clean removal was reflected in our finding. An easily peeled off patch would leave residue and provide lower tacking strength (F2), possibly due to the lower polymer content resulting in less cohesion and adhesion force. This also implied to the result for F4. Therefore, F3 was found to be the most appropriate formulation for PSA hydrogel with ideal characteristics and chosen for further investigation on the drug release [48].

**Figure 6:**
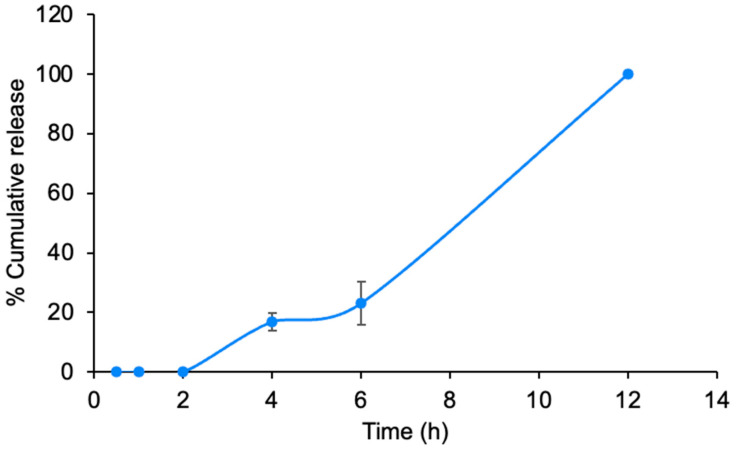
Release profile of F3 hydrogel adhesive over 12 h. The data are expressed as mean ± SD (n = 3).

The drug release from the hydrogel path was conducted to demonstrate the ability of the RAL-FLPs to be released from the PSA hydrogel. After incorporating F3 hydrogel with RAL-FLPs, the release profile shown in Figure 6 demonstrates a biphasic pattern with an initial lag phase followed by a rapid increase in release. During the first two hours, minimal release occurs, indicating that the compound is initially retained within the PSA hydrogel matrix. This could be attributed to slow diffusion or the need for environmental triggers such as hydration or temperature changes to initiate the release process. After 2 h, the release begins to increase gradually, reaching approximately 20%. This slow-release phase suggests a controlled diffusion of the compound out of the matrix or delivery system, possibly due to the presence of barriers that delay release. A significant change is observed after 6 h, where the release rate accelerates sharply, with cumulative release reaching 100% by 12 h. This rapid release phase likely results from the swelling and erosion of the matrix, which triggers the rapid diffusion of the remaining compound. Such a release profile can offer therapeutic advantages, allowing for prolonged and sustained delivery of the active compound, potentially reducing dosing frequency and improving patient compliance. This result suggested an effective drug delivery system using the hydrogel adhesive and proved that the RAL-FLPs can be diffused across the polymeric matrix.

#### 3.2.2. Skin Deposition Study

**Figure 7:**
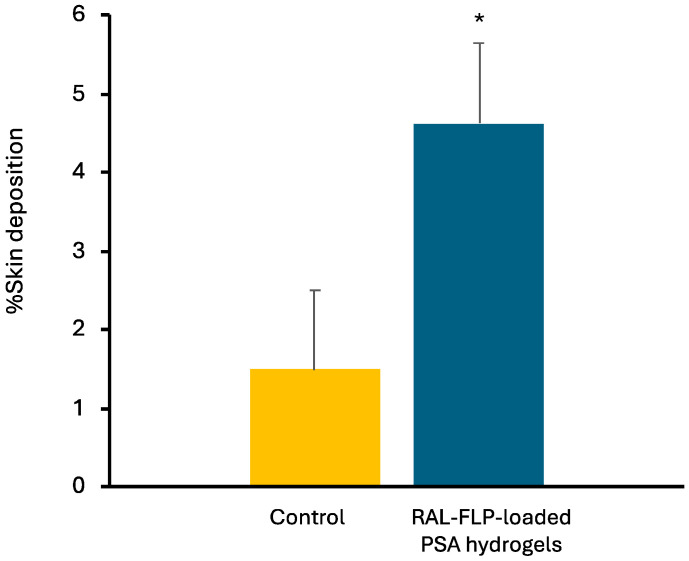
Skin deposition of RAL after 24 h treatment of (■) RAL aqueous solution (control) and (■) RAL LPs in hydrogels. (* significant difference vs control, *p* < 0.05).

After 24 h of applying RAL in different formulations, skin samples were obtained and subjected to analysis for drug accumulation. The data presented in Figure 7 indicates that the RAL-FLP-embedded PSA hydrogel formulation resulted in a significantly greater deposition of RAL in the skin compared with the aqueous solution (used as control). The hydrogels increase the accumulation of the drug in the skin by approximately three times in comparison to the control. The FLP formulation likely improves the solubility and stability of RAL, enabling more efficient delivery across the skin barrier. FLPs, known for their ability to merge with biological membranes due to their phospholipid composition, may facilitate deeper skin penetration, enhancing the delivery of RAL [49]. Additionally, the hydrogel matrix may serve to prolong skin contact time, maintain hydration, and provide a controlled-release mechanism, further boosting RAL permeation. This finding suggested that the RAL-FLP formulation could improve therapeutic efficacy in dermatological applications, such as anti-aging or acne treatments, where enhanced skin permeation and deeper delivery of RAL are preferred [50].

Numerous methods have been employed to enhance the penetration of drugs through the skin. These methods involve chemical enhancers, nanotechnology, energy-based techniques, and stratum corneum bypass or modification. Transdermal patches are well-known for their convenience and non-invasive nature and can alter the structure of the stratum corneum without causing harm. Moreover, transdermal patches can produce an occlusive effect that enhances skin hydration, enabling more effective penetration of the drug into the stratum corneum [51]. Due to high water content, hydrogels can increase stratum corneum hydration. Marwah et. al. (2016) also suggest that hydrogels can induce structural changes in the stratum corneum and improve drug permeability [52]. Previous research by Tan et. al. (2010) demonstrated that after 6 h of hydration, the lipid bilayer of the stratum corneum separates, significantly increasing porcine skin permeability [53].

#### 3.2.3. In Vivo Skin Irritation Study and Adhesion Capability in Human Subjects

**Figure 8:**
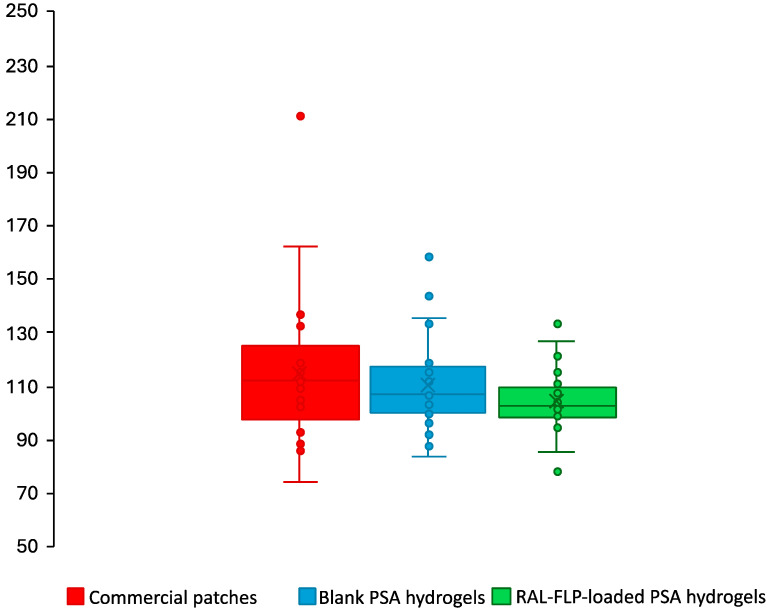
The percentage of erythema index (%EI) of skin after 24 h application of the (■) commercial patches, (■) developed blank PSA hydrogels, and (■) RAL-FLP-embedded PSA hydrogels in 25 participants.

Transdermal patches have gained popularity for drug delivery due to their user-friendly application and non-invasive nature. Despite their advantages, they can lead to various skin reactions, including irritation, contact dermatitis, and allergic contact dermatitis. The occlusion effect of the patches can further increase the risk of sensitization. In addition, excipients in the adhesives, which help the patch stick to the skin, may play a significant role in provoking these allergic or sensitizing responses [54]. The results presented in Figure 8 reveal that after a single 24 h application of both commercial patches and developed hydrogels (blank PSA hydrogels and RAL-FLP-embedded PSA hydrogels), there were no significant changes from baseline in EI values in participants.

Topical retinoids are known to cause skin irritation and dermatitis. RAL, a derivative of retinoids, exhibits a lower skin irritation profile compared to retinol [1]. Nonetheless, local adverse reactions have been reported in certain clinical studies [55]. The incorporation of active ingredients that tend to cause skin irritation in nanotechnology-based formulations is an approach to minimize skin irritation [10,11,12]. According to our results, the comparative analysis revealed no notable differences in the percentage of skin EI across all three patch types (commercial, blank PSA hydrogel, and RAL-FLP-embedded PSA hydrogel) after the application. Furthermore, no participants experienced any visible skin reactions such as rash, itching, hives, or swelling, and there were no abnormal dermal responses. Importantly, no serious adverse effects were reported during the study, indicating a favorable safety profile. These findings suggest that the RAL-LP-loaded hydrogels could be a viable option for transdermal drug delivery, offering the potential for safe and effective therapeutic use.

**Figure 9:**
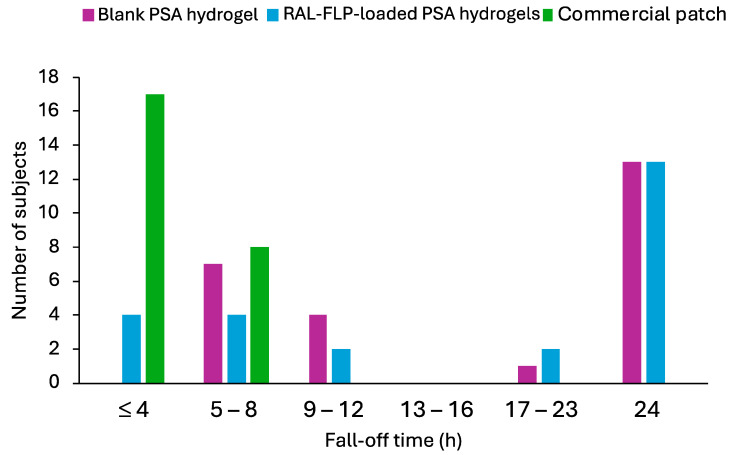
The fall-off time of the (■) commercial patches, (■) blank PSA hydrogels, and (■) RAL-FLP-loaded PSA hydrogels in 25 participants.

Figure 9 illustrates the fall-off times for commercial patches, blank PSA hydrogels, and RAL-FLP-embedded PSA hydrogel. The commercial patches detached within 8 h in all participants. The newly developed PSA hydrogels, both with and without RAL-FLPs, exhibited prolonged adherence, which remain attached for up to 24 h in 13 out of 25 participants (52%). This suggests that drug loading did not have a significant impact on adhesion. Some participants who experienced detachment before 24 h reported that moisture from sweating or showering reduced the PSA hydrogels’ stickiness. Since the hydrogels were made from hydrophilic polymers, exposure to water would cause swelling and reduced adhesion [56]. Despite this, the developed hydrogels demonstrated superior adhesive performance compared to commercial patches, maintaining attachment for up to 24 h, suggesting their potential use in transdermal drug delivery with superior adhesion to the commercially available adhesion patches.

## 4. Conclusions

The development of RAL-FLP infused with α-TS embedded into PSA hydrogels has proven to be a promising strategy for enhancing the stability, skin permeability, and therapeutic efficacy of RAL. The inclusion of α-TS significantly improved the stability of RAL by providing protection and enhancing the fluidity and flexibility of the liposomal bilayer, which in turn increased skin permeation, particularly through the transappendageal route. Additionally, the PSA hydrogels demonstrated excellent adhesive properties, ensuring prolonged skin contact, which further enhanced the drug’s permeation and retention in the skin. In vitro studies confirmed that RAL-FLPs achieved superior skin penetration compared to conventional LP and RAL aqueous solutions, while human safety study demonstrated that the hydrogel formulation was well tolerated, with minimal skin irritation and strong adhesive performance. These findings highlight the potential of RAL-FLP-embedded PSA hydrogels as a safe and effective delivery platform for topical applications, particularly in dermatological treatments requiring controlled and enhanced delivery of the active ingredient.

Future research should explore further optimization of the liposomal and hydrogel compositions to improve permeation efficiency, as well as clinical evaluations to establish long-term efficacy and safety in patients with various skin conditions. Moreover, the long-term stability of the formulation could be investigated to establish its potential for commercial applications. The versatility of this delivery system also opens possibilities for incorporating other lipophilic compounds for targeted transdermal therapies.

## Figures and Tables

**Table 1:** The formulations of GT/Hya PSA patches.

Formulations	Amount (%*w*/*w*)					
	Gantrez^TM^ S-97	Hya	Al Glycinate	Malic Acid	Glycerin	Water q.s. to
F1	20	1	0.1	0.02	20	100
F2	15	1	0.1	0.02	20	100
F3	25	1	0.1	0.02	20	100
F4	20	0.5	0.1	0.02	20	100
F5	20	2	0.1	0.02	20	100

**Table 2:** Characterization of liposomal formulations.

Formulations	Particle Size (nm) ^†^	PDI	ZP (mV)
α-tocopherol LPs	55.11 ± 0.51	0.29 ± 0.01	–27.70 ± 1.44
Conventional LPs	80.71 ± 0.76	0.21 ± 0.01	–4.78 ± 0.44

^†^ Particle size averaged by intensities.

**Table 3:** Physicochemical characteristics and RAL content of RAL-FLP formulations (significant difference compared to R2 (*) and R3 (^#^), *p* < 0.05).

RAL-FLP Formulation	Initial RAL Concentration (mg/mL)	Particle Size (nm)	PDI	ZP (mV)	%LC	%EE
R1	1	46.14 ± 1.97	0.26 ± 0.01	–36.32 ± 1.78	55.42 ± 3.61 *^#^	52.68 ± 3.43 *^#^
R2	3	59.46 ± 0.54	0.33 ± 0.01	–37.64 ± 6.87	42.71 ± 1.80 ^#^	16.38 ± 0.69 ^#^
R3	5	71.48 ± 0.47	0.26 ± 0.02	–39.50 ± 4.91	38.26 ± 2.07 *	10.33 ± 0.56 *

**Table 4:** Tacking strength and peeling adhesion force of pressure-sensitive adhesives (* Significant different compared to F3, *p* < 0.05).

Formulations	Appearance	Tacking Strength (N)	Peeling Force (N)
F1	Soft and adhesive	2.44 ± 0.21 *	3.44 ± 0.50
F2	Leave residue	3.05 ± 0.01 *	0.92 ± 0.02 *
F3	Soft and adhesive	5.73 ± 0.30	3.13 ± 0.90
F4	Leave residue	4.01 ± 0.67 *	2.22 ± 0.36
F5	Leave residue	6.37 ± 0.63	3.80 ± 0.90

## Data Availability

Data available on request due to restriction.
